# RhoJ interacts with the GIT–PIX complex and regulates focal adhesion disassembly

**DOI:** 10.1242/jcs.140434

**Published:** 2014-07-15

**Authors:** Eleanor Wilson, Katarzyna Leszczynska, Natalie S. Poulter, Francesca Edelmann, Victoria A. Salisbury, Peter J. Noy, Andrea Bacon, Joshua Z. Rappoport, John K. Heath, Roy Bicknell, Victoria L. Heath

**Affiliations:** 1 School of Immunity and Infection, Institute for Biomedical Research, The Medical School, University of Birmingham, Birmingham B15 2TT, UK; 2 School of Biosciences, University of Birmingham, Birmingham B15 2TT, UK

**Keywords:** RhoJ, Focal adhesion, GIT, PIX, Angiogenesis

## Abstract

RhoJ is a Rho GTPase expressed in endothelial cells and tumour cells, which regulates cell motility, invasion, endothelial tube formation and focal adhesion numbers. This study aimed to further delineate the molecular function of RhoJ. Using timelapse microscopy RhoJ was found to regulate focal adhesion disassembly; small interfering RNA (siRNA)-mediated knockdown of RhoJ increased focal adhesion disassembly time, whereas expression of an active mutant (daRhoJ) decreased it. Furthermore, daRhoJ co-precipitated with the GIT–PIX complex, a regulator of focal adhesion disassembly. An interaction between daRhoJ and GIT1 was confirmed using yeast two-hybrid experiments, and this depended on the Spa homology domain of GIT1. GIT1, GIT2, β-PIX (also known as ARHGEF7) and RhoJ all colocalised in focal adhesions and depended on each other for their recruitment to focal adhesions. Functionally, the GIT–PIX complex regulated endothelial tube formation, with knockdown of both GIT1 and GIT2, or β-PIX phenocopying RhoJ knockdown. RhoJ-knockout mice showed reduced tumour growth and diminished tumour vessel density, identifying a role for RhoJ in mediating tumour angiogenesis. These studies give new insight into the molecular function of RhoJ in regulating cell motility and tumour vessel formation.

## INTRODUCTION

Cell motility is fundamental to numerous developmental and physiological processes, as well as being crucial in the pathogenesis of diseases such as cancer where it is a pre-requisite for the metastasis of tumours to distant organs. Cell movement depends on the remodelling of the cytoskeleton and coordinated modulation of contacts with the extracellular matrix. Central to the regulation of the actin cytoskeleton are Rho GTPases, molecular switches which orchestrate its dynamic rearrangement to enable cell movement (Sit and Manser, 2011).

RhoJ belongs to the Cdc42 subfamily of Rho GTPases, and cycles between active GTP-bound and inactive GDP-bound forms (Burridge and Wennerberg, 2004). It was first identified in 2000 (Vignal et al., 2000) and early studies suggested roles for RhoJ in modulating the actin cytoskeleton, early endocytosis and adipocyte differentiation (Abe et al., 2003; Aspenström et al., 2004; de Toledo et al., 2003; Nishizuka et al., 2003; Vignal et al., 2000). Subsequently it was found to be expressed in endothelial cells (Fukushima et al., 2011; Kaur et al., 2011; Takase et al., 2012; Yuan et al., 2011) and induced by the transcription factor Erg (Yuan et al., 2011). Functionally, RhoJ has been shown to regulate endothelial motility, tubulogenesis and lumen formation *in vitro* (Kaur et al., 2011; Yuan et al., 2011) and vascularisation *in vivo* (Kim et al., 2014; Takase et al., 2012; Yuan et al., 2011). Recently, a role for RhoJ has been identified in regulating the motility and invasion of melanoma cells, suggesting a role for RhoJ in the metastatic spread of malignant melanoma (Ho et al., 2013). Reducing RhoJ expression using small interfering RNA (siRNA) is associated with an impairment in motility (Ho et al., 2013; Kaur et al., 2011), and this in turn is associated with increased actinomyosin contractility (Kaur et al., 2011). This increase in contractility is consistent with observations that RhoJ knockdown causes decreased levels of active Rac and Cdc42 and increased levels of active RhoA and phosphorylated myosin light chain (Kaur et al., 2011; Yuan et al., 2011). RhoJ has been found to both localise to focal adhesions and to regulate their numbers (Kaur et al., 2011). These adhesions connect the intracellular actin cytoskeleton to the extracellular matrix through integrins, which are transmembrane proteins, and the coordinated assembly and disassembly of focal adhesions are crucial to cell motility (Parsons et al., 2010).

The GIT–PIX complex is an oligomeric protein assembly that acts as a scaffold and signal integrator (Frank and Hansen, 2008; Hoefen and Berk, 2006). Within focal adhesions, it functions to regulate their maturation and disassembly (Feng et al., 2010; Kuo et al., 2011; Nayal et al., 2006; Zhao et al., 2000). There are two G-protein-coupled receptor kinase-interacting target (GIT) proteins, GIT1/CAT-1 and GIT2/CAT-2/PKL (Bagrodia et al., 1999; Di Cesare et al., 2000; Premont et al., 1998; Turner et al., 1999), and two Pak-interacting exchange factor (PIX) proteins, α-PIX (also known as ARHGEF6 and Cool-2) and β-PIX (also known as ARHGEF7 and Cool-1) (Bagrodia et al., 1998; Manser et al., 1998; Oh et al., 1997). Both GIT and PIX proteins have multiple domains and interacting partners. GIT proteins are recruited to focal adhesions through their binding of paxillin (Di Cesare et al., 2000; Turner et al., 1999; Zhao et al., 2000) and have ARF-GAP activity which is likely involved in their trafficking and localisation (Di Cesare et al., 2000; Matafora et al., 2001). GIT proteins associate through their Spa homology domains (SHD) with PIX proteins (Premont et al., 2004; Zhao et al., 2000), which in turn results in the recruitment of the kinase PAK to focal adhesions through its binding to PIX. In addition, PIX proteins contain Cdc42 and Rac guanine-nucleotide-exchange factor (GEF) domains (Bagrodia et al., 1998; Manser et al., 1998). A number of studies indicate that promoting the localisation of the PAK–PIX–GIT complex to focal adhesions increases cellular motility and protrusions (Manabe et al., 2002; West et al., 2001; Zhao et al., 2000).

The purpose of this study was to characterise the molecular mechanism by which RhoJ modulates focal adhesion dynamics and determine its role in angiogenesis *in vivo*. RhoJ was found to regulate focal adhesion disassembly and the active form of RhoJ interacted with the GIT–PIX complex in pulldown experiments. Yeast two-hybrid experiments also identified that active RhoJ interacted with the SHD of GIT1. RhoJ, GIT1, GIT2 and β-PIX all potentiated the recruitment of each other to focal adhesions. Like RhoJ, a role for β-PIX, GIT1 and GIT2 in endothelial tube formation was discovered. Knockout of RhoJ reduced the growth and vascularisation of subcutaneous tumours compared with wild-type controls. This study demonstrates a role for RhoJ in regulating tumour angiogenesis and in mediating focal adhesion dynamics through its association with the GIT–PIX complex.

## RESULTS

### RhoJ regulates focal adhesion disassembly

**Fig. 1.**
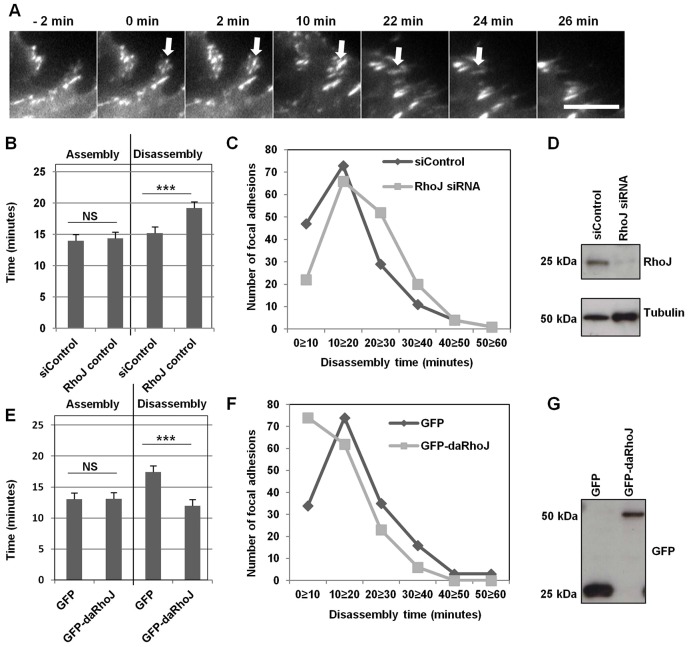
**RhoJ regulates focal adhesion disassembly in endothelial cells.** (A) HUVECs expressing paxillin–RFP at a scratch edge were imaged every 2 minutes by TIRF microscopy and focal adhesions were tracked. Representative images show a focal adhesion (arrow) appearing at time 0 and disappearing after 26 minutes. Assembly times were the time from first appearance to maximal intensity and disassembly times were the time from the maximal intensity to disappearance. Scale bar: 10 µm. (B) Paxillin–RFP-expressing HUVECs were transfected with either control siRNA (siControl) or RhoJ siRNA duplex 48 hours before imaging. Assembly and disassembly times were measured from 10–15 adhesions from each of 11 cells (three or four cells from three independent experiments, a total of 155 adhesions per condition) and displayed as a bar chart (mean±s.e.m.). (C) Data from B were also plotted as a histogram showing the number of focal adhesions with different disassembly times. (D) Reduction in RhoJ expression was confirmed by western blotting with tubulin as a loading control. (E,F) Paxillin–RFP-expressing HUVECs were transduced to express either GFP or GFP–daRhoJ, and focal adhesion assembly and disassembly times were measured as above, from 10–15 adhesions from each of 12 cells (four cells from three independent experiments, a total of 165 adhesions per condition) and displayed as a bar chart (mean±s.e.m.) (E) and a histogram (F). (G) Expression of GFP and GFP–daRhoJ was confirmed with western blotting for GFP. ***P<0.001; NS, not significant (Mann–Whitney test).

RhoJ had previously been shown to regulate both cell motility and focal adhesion number. A reduction in the level of RhoJ leads to reduced motility and increased focal adhesion numbers, whereas the expression of an active GTP-bound mutant (daRhoJ) results in fewer focal adhesions and increased motility. In order to more precisely determine how RhoJ affects focal adhesion dynamics, experiments were performed to track the assembly and disassembly of focal adhesions in endothelial cells manipulated for their expression and activity of RhoJ. To investigate reduced RhoJ activity, siRNA knockdown rather than a dominant-negative mutant was used to specifically reduce levels of this Rho GTPase; the promiscuous binding of GEF proteins to multiple related Rho GTPases would be likely to result in a dominant-negative mutant of RhoJ sequestering and inhibiting GEFs of Cdc42 or Rac (Debreceni et al., 2004; Schmidt and Hall, 2002). In order to track focal adhesions, human umbilical vein endothelial cells (HUVECs) were transduced with an RFP-tagged paxillin and subjected to total internal reflection fluorescence (TIRF) microscopy, which is suited for visualisation of structures close to the cell surface (Mattheyses et al., 2010). Paxillin is a well-characterised focal adhesion protein and this fusion has been previously used for studying focal adhesion dynamics (Berginski et al., 2011). In order to confirm its suitability for these studies, it was confirmed that siRNA-mediated RhoJ knockdown increased numbers of paxillin–RFP-positive focal adhesions, and that expressing paxillin–RFP did not affect numbers of focal adhesions (data not shown). Paxillin–RFP-transduced HUVECs were treated with either a siRNA control duplex or a RhoJ-specific duplex, plated at a high cell density and then the monolayer was scratched. Motile cells at the edge of the scratch wound, where the focal adhesion phenotype is evident, were then tracked over a period of 90 minutes, imaging every 2 minutes. A representative series of images is shown in Fig. 1A. The duration times of focal adhesions in cells from each condition were manually tracked and considered to be the time taken from their first appearance to when they were no longer visible. The fluorescence intensity was also measured to enable assembly and disassembly times to be calculated; the assembly time being the time from appearance to the peak fluorescence and the disassembly time being from the peak to disappearance of the focal adhesion. It was found that RhoJ knockdown resulted in significantly longer focal adhesion duration times (data not shown). When this was broken down into assembly and disassembly times, it was found that the disassembly times, but not the assembly times, were significantly longer after knockdown of RhoJ (Fig. 1B). Displaying the data as a histogram, with the number of focal adhesions disassembling during 10-minute intervals shows that siRNA knockdown of RhoJ results in a shift to the right with higher numbers of focal adhesions taking more than 20 minutes to disassemble (Fig. 1C). Levels of RhoJ knockdown were confirmed by western blot with tubulin as a loading control (Fig. 1D).

To look at the effect of increased RhoJ activity on focal adhesions, HUVECs were transduced to express both paxillin–RFP and either GFP or GFP-tagged daRhoJ (GFP–daRhoJ). Focal adhesions in cells at the edge of a scratch were monitored as described above. In contrast to reducing expression of RhoJ, introduction of active RhoJ resulted in faster focal adhesion turnover and again it was disassembly rather than assembly that was changed (Fig. 1E). Expression of GFP–daRhoJ resulted in a statistically significant reduction in disassembly times compared with the GFP control, and plotting the data as a histogram resulted in a shift to the left with the majority of focal adhesions disassembling in less than 20 minutes (Fig. 1F). Western blots showed that there was substantial expression of both GFP and the GFP–daRhoJ fusion proteins (Fig. 1G). These data demonstrate that RhoJ activity promotes focal adhesion disassembly, and is consistent with our previous observations that RhoJ regulates focal adhesion numbers (Kaur et al., 2011): RhoJ knockdown causing increased numbers of focal adhesions owing to slower disassembly, and daRhoJ causing decreased numbers owing to more rapid disassembly.

### RhoJ interacts with the GIT–PIX complex

In order to better characterise the molecular basis for the role of RhoJ in focal adhesion disassembly, experiments were performed to determine its interacting partners. Pulldown experiments were performed using recombinant glutathione S-transferase (GST)-tagged daRhoJ incubated with HUVEC lysate and candidate interacting proteins were identified using mass spectrometry. Two candidate interacting proteins were the focal adhesion proteins GIT1 (11 peptides, 4.8% coverage) and β-PIX (53 peptides, 6.9% coverage). These proteins were of particular interest given the role of the GIT–PIX complex in focal adhesion disassembly (Feng et al., 2010; Kuo et al., 2011; Nayal et al., 2006; Zhao et al., 2000). GIT2 and α-PIX are structurally related to GIT1 and β-PIX, respectively. Levels of GIT2 mRNA were found to be higher than those of GIT1 in HUVECs, while β-PIX was expressed at a higher level than α-PIX in this cell type (data not shown). Given its similarity to GIT1, GIT2 was also explored as a potential interacting partner for RhoJ.

**Fig. 2.**
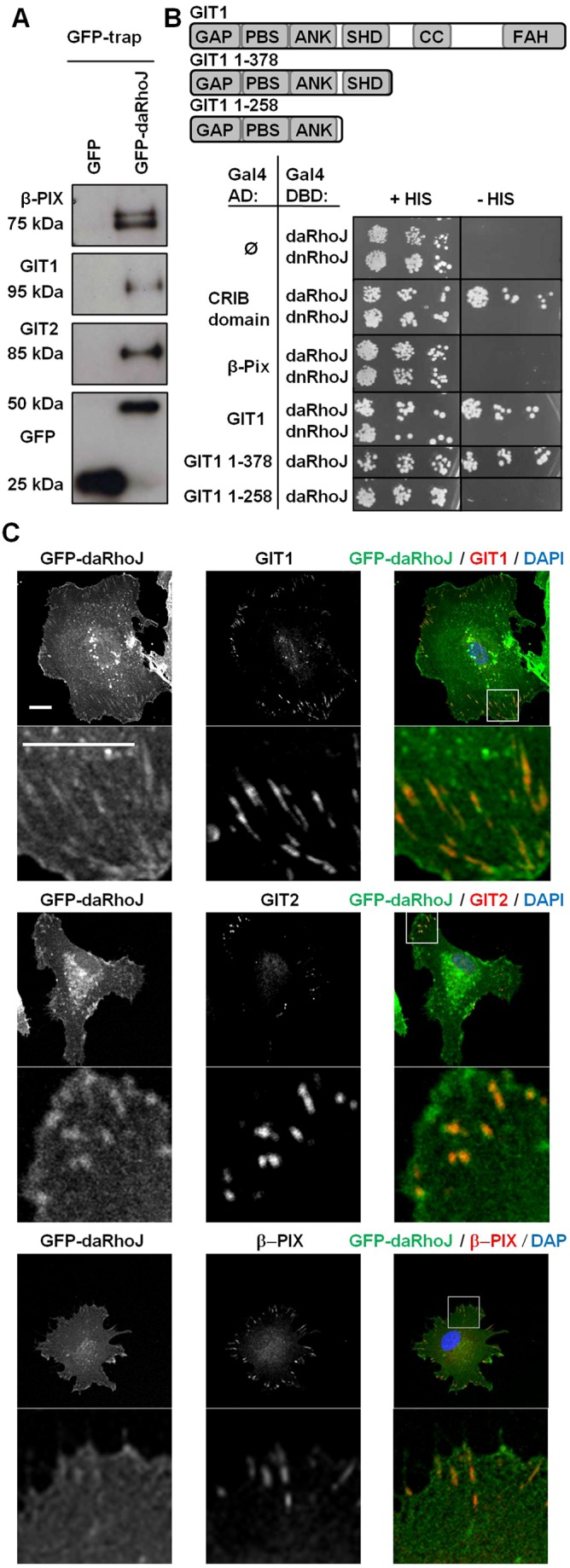
**RhoJ interacts and colocalises with β-PIX, GIT1 and GIT2.** (A) HUVECs stably expressing GFP or GFP–daRhoJ were lysed and pulldowns performed using GFP-trap beads. Samples were probed by western blot for interactions with β-PIX, GIT1 and GIT2, using GFP as a binding control. (B) Yeast two-hybrid assays were performed using yeast transformed with Gal4 activation domain (AD) fusions of candidate interacting partners and Gal4 DNA-binding domain (DBD) fusions of daRhoJ or dnRhoJ. Positive interactions are indicated by growth of yeast on plates lacking Histidine (–His). Truncations of GIT1 were performed to map its interaction with RhoJ, with the Spa homology domain (SHD) found to be necessary for this binding. (C) HUVECs transduced to express GFP–daRhoJ were fixed and stained for GIT1, GIT2 and β-PIX. The box indicates the enlarged area. Scale bar: 20 µM. These data are representative of three independent experiments.

In order to confirm these interactions, cellular lysates were prepared from HUVECs expressing either GFP or GFP–daRhoJ, and incubated with GFP-trap beads. Western blotting revealed that β-PIX, GIT1 and GIT2 all co-immunoprecipitated with GFP–daRhoJ, but not with GFP alone (Fig. 2A). To further characterise the interaction of RhoJ with the GIT–PIX complex, yeast two-hybrid was performed to investigate whether either or both of these proteins were able to interact with RhoJ in this system. In order to do this a GAL4 DNA-binding domain fusion with daRhoJ and dominant-negative (dn)RhoJ was constructed. This was co-expressed in yeast with GIT1 or β-PIX fused to the GAL4 activation domain. In the yeast strain used, close interaction of these GAL4 domains results in the transactivation of the GAL1-promoter-driven HIS3 gene enabling this auxotrophic yeast strain to grow on plates lacking histidine. These assays showed that daRhoJ, but not dnRhoJ, interacted with GIT1 but not β-PIX to allow growth on medium lacking histidine (Fig. 2B). A series of C-terminal truncation mutants of GIT1 were created to determine which regions of GIT1 were necessary for interaction with RhoJ, these identified a region including the SHD as being necessary (Fig. 2B). Both GIT1 and GIT2 have SHDs with a sequence identity of 90% and 87% for SHD1 and SHD2, respectively, with non-identical amino acids being similar, and so it is likely that daRhoJ would also bind this region of GIT2. These data suggest that GIT1 and most likely GIT2 bind daRhoJ through their SHDs, and β-PIX is co-immunoprecipitated with daRhoJ through its association with GIT1 and/or GIT2. Endogenous GIT1, GIT2, RhoJ and β-PIX all colocalised with vinculin at focal adhesions (supplementary material Fig. S1A), and there was colocalisation of GFP–daRhoJ with GIT1, GIT2 and β-PIX at focal adhesions (Fig. 2C).

### RhoJ regulates focal adhesion size

**Fig. 3.**
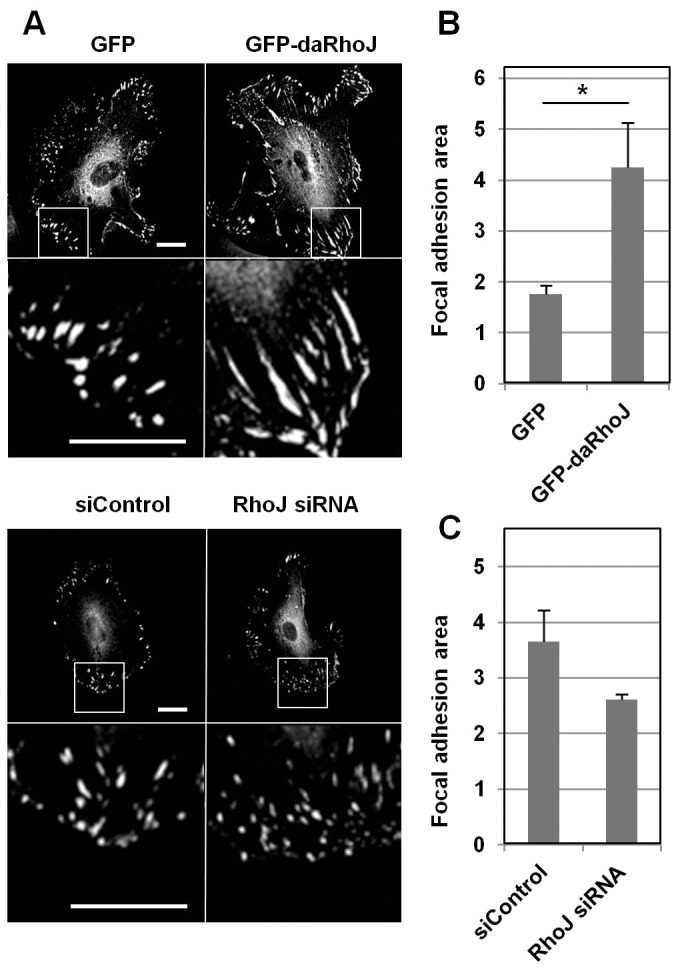
**RhoJ regulates focal adhesion size.** (A) HUVECs were transfected with control siRNA (siControl) or RhoJ siRNA duplexes and after 48 hours were stained with vinculin-specific antibodies. Similarly, HUVECs transduced to express GFP or GFP–daRhoJ were fixed and stained for vinculin. The box indicates the enlarged area. Scale bar: 20 µm. (B) Focal adhesion areas from GFP- and GFP–daRhoJ-expressing HUVECs were measured using ImageJ of 15 focal adhesions per cell from a total of three cells, and the mean focal adhesion area calculated for each condition. This was performed three times and plotted are the mean values from each experiment (*n* = 3) (mean±s.e.m.). *P<0.05 (Student's *t*-test). (C) Similarly, focal adhesion areas from HUVECs transfected with siControl or RhoJ siRNA were measured using ImageJ of 106–135 focal adhesions from a total of five or six cells and mean focal adhesion area calculated per condition, plotted are the mean focal adhesion areas from three independent experiments (*n* = 3) (mean±s.e.m.). Reductions in focal adhesion areas were observed in each of the three experiments.

As well as regulating focal adhesion numbers (Kaur et al., 2011), during the course of this study we observed that the activity of RhoJ within endothelial cells also affected the size of focal adhesions. HUVECs expressing either GFP or GFP–daRhoJ were fixed and immunofluorescence staining of vinculin was performed (Fig. 3A). The area of vinculin staining was quantified for at least 15 focal adhesions randomly selected from three cells and the mean focal adhesion area for each condition calculated. Data from three replicate experiments showed a statistically significant increase in focal adhesion size in HUVECs expressing GFP–daRhoJ compared those expressing GFP (Fig. 3B). In contrast, HUVECs transfected with two different RhoJ-specific siRNA duplexes were found to consistently have a small reduction in focal adhesion area compared with control siRNA-transfected HUVECs across three experiments (Fig. 3A,C; supplementary material Fig. S1B,C).

**Fig. 4.**
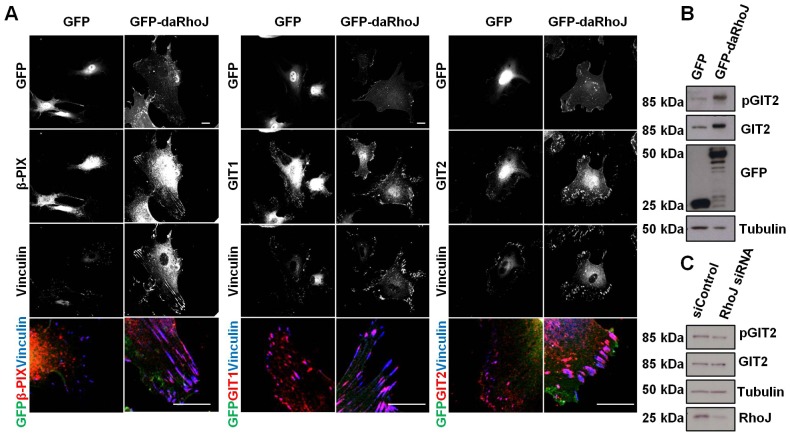
**GFP–daRhoJ expression increases recruitment of partner proteins to focal adhesions.** (A) HUVECs were transduced to express GFP or GFP–daRhoJ, and were fixed and stained for vinculin and either β-PIX, GIT1 or GIT2. Scale bar: 20 µm. (B) Cellular lysates were prepared from HUVECs expressing GFP or GFP–daRhoJ and western blotted for GIT2 phosphorylated on Y392 (pGIT2), GIT2, GFP and tubulin. (C) HUVECs were transfected with control siRNA (siControl) or RhoJ siRNA duplexes. After 48 hours, cells were lysed and blotted for GIT2 phosphorylated on Y392, GIT2, RhoJ and tubulin. These data are representative of three independent experiments.

Consistent with these observations was the increase in levels of GIT1, GIT2 and β-PIX found at focal adhesions in GFP–daRhoJ-expressing cells compared with those expressing GFP alone (Fig. 4A). In the case of GIT2 we observed higher levels of GIT2 protein in lysates from HUVECs expressing GFP–daRhoJ, compared with control GFP-expressing cells. In order for its recruitment to focal adhesions, GIT2 must be tyrosine phosphorylated by Src and focal adhesion kinase (FAK) (Brown et al., 2005). Levels of phospho-GIT2 (Y392) were also found to be elevated to the same extent as total GIT2 in GFP–daRhoJ-expressing cells (Fig. 4B). Increased recruitment of GIT2 to focal adhesions through interaction with daRhoJ might result in its stabilisation and protection from degradation. There is also a shift in size of GIT2 from HUVECs expressing daRhoJ, this is likely to be due to increased levels of serine and threonine phosphorylation because phosphorylation of these residues in GIT1 has been observed (Webb et al., 2006b). Inhibition of Src and FAK reduced levels of phospho-GIT2 (Y392), but did not change its electrophoretic mobility (supplementary material Fig. S2). Knockdown of RhoJ did not lead to lower levels of GIT2 protein, but rather resulted in reduced levels of GIT2 phosphorylation (Fig. 4C), and although the reduction was small it was consistently observed. The reduced level of phosphorylation might be due to reduced GIT2 being recruited to focal adhesions (supplementary material Fig. S3D) and being phosphorylated there.

### RhoJ, GIT1, GIT2 and β-PIX are involved in recruitment of each other at focal adhesions

**Fig. 5.**
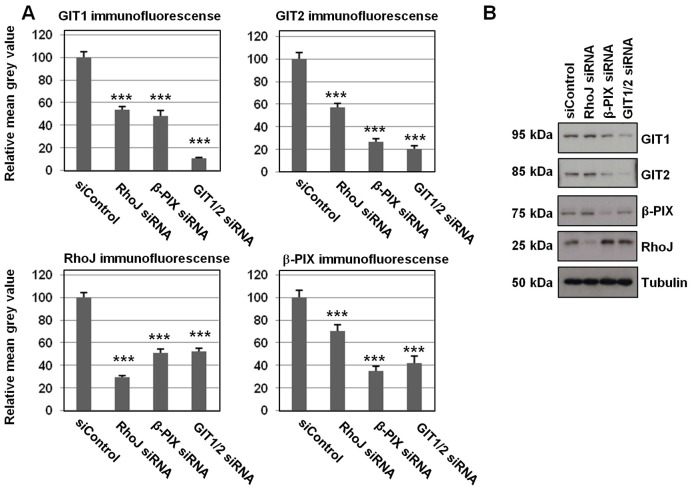
**Reciprocal regulation of the recruitment of RhoJ, GIT1/2 and β-PIX to focal adhesions.** (A) HUVECs were transfected with control siRNA (siControl), RhoJ siRNA, β-PIX siRNA or both GIT1 and GIT2 (GIT1/2) siRNA duplexes. After 48 hours, HUVECs were fixed and stained for vinculin, and RhoJ, β-PIX, GIT1 or GIT2. For each experiment, the mean grey value of either GIT1, GIT2, RhoJ or β-PIX staining (as indicated) was calculated for 20 adhesions per cell from three cells using ImageJ from three independent experiments according to the Materials and Methods. For each replicate experiment, all data points were scaled to the mean of the siControl, which was set at 100. Plotted are the means of all the scaled data points from each condition from each of the experimental replicates (mean±s.e.m.). ***P<0.001 (Mann–Whitney test comparing each of the data points to the siControl). (B) Cells were lysed after 48 and 72 hours and blotted for GIT1, GIT2, RhoJ, β-PIX or tubulin as a loading control.

Given the physical interactions between GIT1 and GIT2 with β-PIX and RhoJ, experiments were performed to determine whether knocking down expression of each of these components affected the recruitment of the others to focal adhesions. HUVECs were transfected with the siRNA control duplex, duplexes specific for RhoJ, β-PIX or a combination of GIT1 and GIT2 duplexes. Owing to the similarity and redundancy between GIT1 and GIT2 and the fact both are expressed in HUVECs, knockdowns of both GIT1 and GIT2 were performed together. At 2 days after transfection, cells were fixed and then immunofluorescence staining was performed for the focal adhesion protein vinculin in combination with antibodies specific to RhoJ, β-PIX, GIT1 or GIT2. Focal adhesions were identified by their staining for vinculin, and the mean grey value for RhoJ, GIT1, GIT2 or β-PIX staining in the region positive for vinculin was analysed. It was found that knocking down any of these components resulted in a reduction of staining with antibodies specific to any other member of the complex. Examples of the immunofluorescence staining are shown in supplementary material Fig. S3A–D. A total of 20 focal adhesions were examined from each of three cells. In order to compare data generated from different experiments, mean grey values were scaled to the average mean grey value for the siRNA control duplex. Thus for each replicate experiment, data points from each experimental group, including those of the negative control duplex, were scaled to the mean of the negative control data for that experiment which was set at 100. Data from focal adhesions from three independent experiments were then combined and are shown in Fig. 5A. Very similar results were obtained with an alternative set of siRNA duplexes (supplementary material Fig. S3E). Knockdown of RhoJ, GIT1 and GIT2 together, and β-PIX were confirmed at 48 hours by western blotting (Fig. 5B). Thus, knocking down RhoJ resulted in not only a significant decrease in RhoJ localisation to focal adhesions but also that of GIT1, GIT2 and β-PIX. Similarly β-PIX knockdown resulted in reduced GIT1, GIT2 and RhoJ at focal adhesions; likewise knockdown of GIT1 and GIT2 together caused less RhoJ and β-PIX to be recruited to focal adhesions.

### β-PIX, GIT1 and GIT2 regulate tubulogenesis

**Fig. 6.**
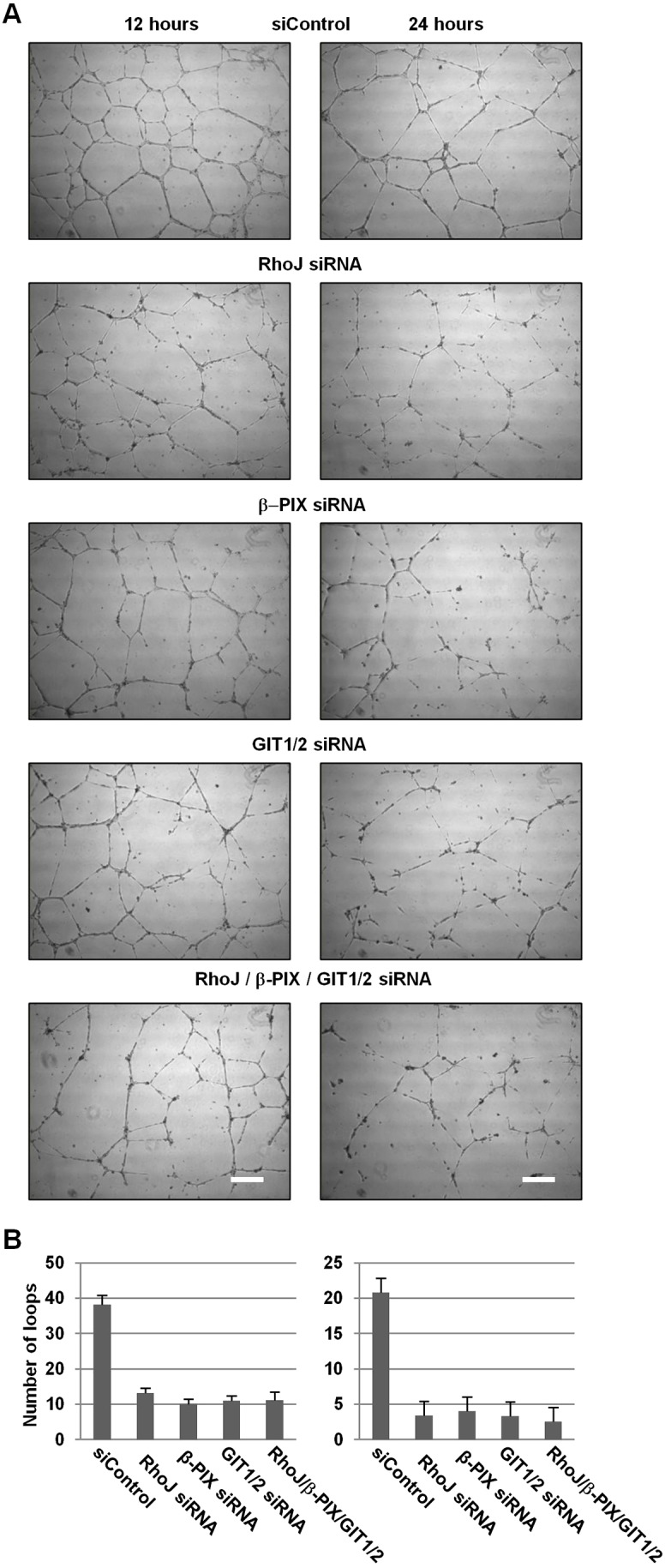
**Knockdown of RhoJ, β-PIX, both GIT1 and GIT2 or a combination of all four similarly impairs tube formation.** (A) HUVECs were transfected with control siRNA (siControl), RhoJ siRNA, β-PIX siRNA or GIT1 and GIT2 siRNA together (GIT1/2) or a combination of RhoJ, β-PIX, GIT1 and GIT2 duplexes. At 48 hours after transfection, the cells were replated on Matrigel and imaged after 12 and 24 hours. Scale bars: 200 µm. (B) Analysis of the tubule formation using the angiogenesis analyser ImageJ plugin to show the number of loops formed by the tubules. For each experiment the mean loop number was calculated from five to six fields of view per time point. The mean of these values from three experimental replicates is plotted (*n* = 3) (mean±sem). All knockdowns give a statistically significant difference compared with the siControl duplex [*P*<0.05 (Student's *t*-test)], but there are no differences between the individual RhoJ, β-PIX, GIT1/2 knockdowns and knockdown of all four in combination.

Previously we and others had demonstrated a role for RhoJ in regulating endothelial migration and tube formation (Kaur et al., 2011; Yuan et al., 2011). Owing to the biochemical and functional interaction between RhoJ and the GIT–PIX complex, the role of RhoJ, GIT1 and GIT2, and β-PIX alone and in combination in tube formation was also assessed using a Matrigel tube forming assay. In this assay, endothelial cells are seeded on to wells coated with Matrigel, a solubilised basement membrane extract from the Engelbreth–Holm–Swarm sarcoma cell which is rich in collagen IV, laminin, heparin sulphate proteoglycans and entactin (Kalluri, 2003). This extract induces the endothelial cells to form tube-like structures, a process dependent on cell attachment, migration and differentiation of endothelial cells and which is considered to represent the differentiation stage of angiogenesis (Kubota et al., 1988; Lawley and Kubota, 1989). HUVECs were transfected with siRNA control duplex or siRNA against RhoJ, β-PIX, a combination of GIT1 and GIT2 or a combination of all four duplexes. After 2 days, cells were harvested and plated on to Matrigel, and imaged after 12 and 24 hours. The images were processed by software that calculated the number of loops for each image, to give an indication of the connectivity of the network (Fig. 6B). Knockdown of β-PIX and both GIT1 and GIT2 resulted in disrupted tube formation, giving rise to less connected and less stable networks of cells, similar to that observed with RhoJ knockdown (Fig. 6). These data suggest that, like RhoJ, the GIT–PIX complex plays a positive role in tube formation. Knocking down the combination of RhoJ, β-PIX, GIT1 and GIT2 was found to impair tube formation to a similar degree as knocking out RhoJ or β-PIX individually or the combination of GIT1 and GIT2 (Fig. 6). This would be consistent with these proteins acting together in the same pathway, rather than in distinct pathways, where an additive and more severe phenotype would be expected. Similar observations were made using an alternative set of duplexes (supplementary material Fig. S4).

### RhoJ regulates tumour angiogenesis

**Fig. 7.**
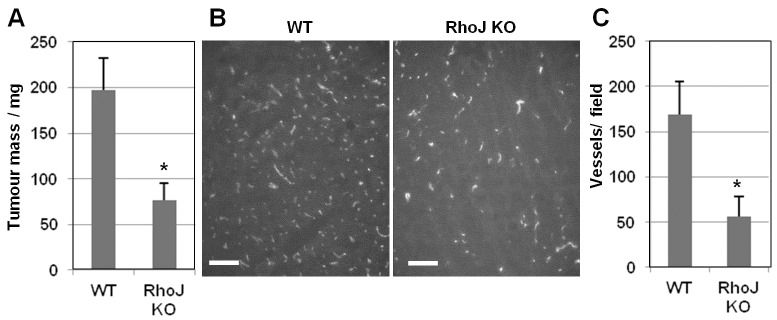
**Reduced tumour growth in RhoJ-knockout mice.** Wild-type (WT) and RhoJ-knockout (KO) mice were subcutaneously implanted with 10^6^ Lewis lung carcinoma cells, after 2 weeks the tumours were excised and weighed (WT, *n* = 5; RhoJ KO *n* = 7). (A) The tumour weights are plotted (mean±s.e.m.). *P = 0.0101 (Mann–Whitney test). (B) The excised tumours were sectioned and vessels stained using anti-CD31 with representative images shown. Scale bar: 100 µm. (C) Vessel density was calculated using the Angiosys Software and plotted (mean±s.e.m.). *P = 0.0303 (Mann–Whitney test).

In order to determine the role of RhoJ *in vivo* a knockout mouse was made. These were derived from embryonic stem cells which contained a gene trap cassette inserted between the first and second exons and which had LoxP sites flanking the second exon. Mice homozygous for the RhoJ genetrap were crossed with mice constitutively expressing Cre recombinase resulting in removal of the second exon. RhoJ-knockout mice were born at the normal Mendelian frequency and grew normally indicating that RhoJ is not essential for embryonic development. However, subcutaneous implantation of syngeneic Lewis lung carcinoma cells resulted in the formation of smaller tumours compared with those in wild-type controls after 2 weeks (Fig. 7A). The rapid growth of these tumours is highly dependent on angiogenesis, the new vessels being required to sustain tumour cell proliferation (Carmeliet and Jain, 2011). To investigate whether tumour vascularisation was affected in the knockout mice, tumours were excised and immunofluorescence staining of the endothelial protein CD31 was performed on frozen sections (Fig. 7B), analysis of vessel density indicated that this was reduced in tumours from RhoJ-knockout mice (Fig. 7C). These data demonstrate a role for RhoJ in mediating tumour angiogenesis.

## DISCUSSION

**Fig. 8.**
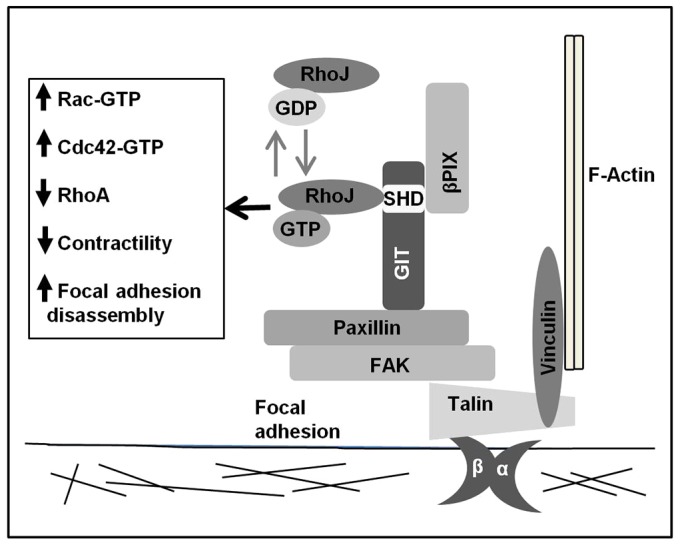
**A model for RhoJ function.** Active RhoJ interacts with the GIT–PIX complex through its interaction with the Spa homology domain (SHD) of GIT1 or GIT2 (GIT). GIT interacts with paxillin and β-PIX. Active RhoJ together with the GIT–PIX complex promote activation of Rac and Cdc42 and focal adhesion disassembly and increased motility. This is associated with decreased RhoA activity and decreased actinomyosin contractility.

We have identified a role for RhoJ in regulating focal adhesion disassembly through its interaction with the GIT–PIX complex and demonstrate that this complex is required for endothelial tube formation. We also show that RhoJ plays a role in tumour angiogenesis *in vivo*. A model for the role of RhoJ within focal adhesions is depicted in Fig. 8. Active GTP-bound RhoJ is localised to focal adhesions and interacts through the SHD of GIT1 and GIT2 to promote the recruitment of the GIT–PIX complex to focal adhesions. This in turn increases the activity of Rac and Cdc42 because of the GEF activity of β-PIX. Although the precise mechanism of GIT–PIX-mediated disassembly has not been delineated, an increased ratio of activated Rac relative to RhoA is associated with adhesion disassembly (Vicente-Manzanares and Horwitz, 2011; Wehrle-Haller, 2012). We found RhoJ, β-PIX and the GIT proteins all promote the recruitment of each other to focal adhesions, and this is likely to be mediated through the interaction between paxillin and GIT1 and/or GIT2. This interaction is potentiated by the PAK-mediated phosphorylation of paxillin (Nayal et al., 2006), thus as the GIT–PIX complex is recruited, the PAK associated with β-PIX is likely to promote further paxillin phosphorylation. Expression of daRhoJ promoted the assembly of larger adhesions. The time taken for these larger adhesions to reach their maximal size was no longer than in the control cells; however, their increased size would indicate an accelerated recruitment of focal adhesion proteins. The faster disassembly of the adhesion would suggest these larger adhesions are less stable. In contrast, knocking down RhoJ expression resulted in smaller more stable adhesions, with reduced levels of the GIT–PIX complex recruited. Our data are consistent with those of Yuan et al. who observed that RhoJ knockdown reduced levels of active Rac and Cdc42 (Yuan et al., 2011).

Previously a number of groups have demonstrated that promoting the localisation of the PAK–PIX–GIT complex to focal adhesions results in increased levels of cellular motility and protrusions (Manabe et al., 2002; West et al., 2001; Zhao et al., 2000). In contrast, knockdown of GIT2 but not GIT1 in epithelial cells was found to decrease focal adhesion size and increase motility, although the effect on focal adhesion turnover times was not measured in this study (Frank et al., 2006). Thus, there are likely to be cell-type- and context-specific variation in the functions of GIT1 and GIT2. Our findings are consistent with work of Kuo et al. who identified a role for β-PIX in driving nascent focal adhesion turnover (Kuo et al., 2011). They found that siRNA knockdown of β-PIX specifically reduced focal adhesion disassembly rather than assembly, mirroring our observations with knockdown of RhoJ. They investigated β-PIX after finding it at increased levels in focal adhesions in cells with reduced actinomyosin contractility induced by blebbistatin-mediated inhibition of myosin II. Previously, we have shown that there is a link between RhoJ activation and contractility in endothelial cells, with RhoJ negatively regulating myosin light chain phosphorylation, actin stress fibre formation and contraction of collagen gels (Kaur et al., 2011). Thus, both myosin II inhibition and daRhoJ expression result in decreased contractility and an associated increased β-PIX accumulation at focal adhesions (Kuo et al., 2011 and Fig. 4A). There is a complex relationship between focal adhesion maturation, turnover and actinomyosin contractility; thus, as myosin II activity can influence focal adhesion size and distribution, so too can focal adhesion proteins regulate contractility (Parsons et al., 2010). Our data would suggest that RhoJ regulates contractility through its interaction and modulation of GIT–PIX-mediated focal adhesion turnover.

The GIT–PIX complex is multimeric. GIT proteins form homodimers and heterodimers through their coiled coil domains (Paris et al., 2003; Premont et al., 2004), likewise similar domains in the C-terminus of β-PIX enable its homodimerisation (Kim et al., 2001; Koh et al., 2001). Recent structural studies have suggested that β-PIX forms trimers rather than dimers, resulting in a heteropentameric complex in which each SHD of GIT1 binds the GIT-binding domains of two of the three β-PIX proteins (Schlenker and Rittinger, 2009). We identified the SHD of GIT as being a region necessary for the binding of daRhoJ. Besides RhoJ and β-PIX, phospholipase Cγ, FAK, MEK and the presynaptic matrix protein piccolo all have been shown to interact with the SHD (Haendeler et al., 2003; Kim et al., 2003; Yin et al., 2004; Zhao et al., 2000). The finding that GIT1 acts as a MEK1 and ERK1/2 scaffold in focal adhesions might explain the reduced levels of active B-Raf in endothelial cells with reduced RhoJ expression (Yin et al., 2005; Yuan et al., 2011). However, data generated using mutagenesis suggested that piccolo, FAK and β-PIX all interact with different regions of the SHD. Our pulldown experiments would suggest that daRhoJ does not compete with β-PIX for GIT binding because daRhoJ was able to pulldown β-PIX, presumably through its interaction with GIT1 or GIT2. It is possible that binding of active RhoJ to GIT1 and/or GIT2 might act to regulate focal adhesion turnover through displacement of another GIT-binding protein. Thus the SHD of GIT mediates multiple interactions, the nature of which is likely to depend on the cell type and activation.

Multiple sites of serine, threonine and tyrosine phosphorylation have been identified on GIT1 (Webb et al., 2006b), with some modulating the binding of interacting proteins, such as paxillin and Vav2 (Jones et al., 2013; Webb et al., 2006a). Expression of daRhoJ results in increased levels of GIT2, which was phosphorylated at Y392, one of three tyrosine residues whose phosphorylation is required for focal adhesion localisation (Brown et al., 2005). The GIT2 from daRhoJ-expressing endothelial cells ran at an increased molecular mass, most likely due to increased serine and threonine phosphorylation. Incubation with Src and/or FAK inhibitors reduced Y392 phosphorylation but did not alter the increased molecular mass of GIT2 from daRhoJ-expressing HUVEC (supplementary material Fig. S3). Co-expression of active Cdc42 and β-PIX causes increased activation of the serine threonine kinase PAK (Manser et al., 1998), and it is possible that daRhoJ is also activating PAK, causing it to phosphorylate GIT2. We did not find increased levels of GIT1 or any change in its size in daRhoJ-expressing cells, suggesting that RhoJ might be preferentially interacting with GIT2.

We find that both GIT1 and GIT2 are expressed in HUVECs, although GIT2 is more readily detectable by RT-PCR (data not shown). Expression analyses suggest widespread expression of GIT2, whereas GIT1 is localised to endothelial cells, cells lining the bronchi and the bile duct (Schmalzigaug et al., 2007). Although GIT1-knockout mice have impaired vascularisation of the developing lung (Pang et al., 2009), no vascular phenotype has been reported for the GIT2 knockout (Schmalzigaug et al., 2009). This might be due to functional redundancy between these two GIT proteins. Roles for the PIX–GIT–PAK complex have been identified in regulating endothelial cell podosome formation, barrier function, contractility and vascular permeability (Shikata et al., 2003; Stockton et al., 2007; van Nieuw Amerongen et al., 2004; Wang et al., 2009). A role for the GIT–PIX complex in mediating vascular stability has also been suggested because there is increased cerebral haemorrhage in developing zebrafish with either β-PIX mutation or knocked down GIT1 expression (Liu et al., 2012). Slit2 is a ligand for the roundabout receptors that promotes vascular stability. Upon binding its ligand, Robo4 interacts with a paxillin–GIT1 complex sequestering GIT1 away from focal adhesions, and impairing cellular protrusive activity (Jones et al., 2009). In this study, we have shown that, like RhoJ, the GIT–PIX complex positively regulates endothelial tube formation. Combined knockdown of RhoJ with the GIT–PIX complex gives a similar level inhibition of tube formation as knocking down RhoJ, β-PIX and GIT alone. These findings are consistent with RhoJ and the GIT–PIX complex acting together in the same pathway. Endothelial quiescence or activation is tightly controlled by the balance of pro- and anti-angiogenic stimuli (Carmeliet and Jain, 2011), and they likely act by appropriately controlling the localisation and activity of signal integrators, such as the GIT–PIX complex.

Consistent with its role in endothelial tube formation *in vitro*, are our observations that knockout of RhoJ results in diminished tumour growth and reduced vessel density. These data indicate a role for RhoJ in tumour angiogenesis *in vivo*. Although, knockout of RhoJ has been reported to affect neonatal retinal vascularisation (Takase et al., 2012), and RhoJ is expressed in the developing mouse vasculature (Kaur et al., 2011), it is not essential for development presumably due to compensation from closely related Rho GTPases expressed by endothelial cells. Recently RhoJ has been identified as part of a tumour angiogenesis signature – one of 20 genes highly upregulated in tumour vessels (Masiero et al., 2013). The rapid growth of tumours is crucially dependent on the development of vessels to sustain the tumour cell proliferation (Carmeliet and Jain, 2011), and our data would suggest that RhoJ is required to facilitate this process. Our data are similar to that recently reported (Kim et al., 2014); they demonstrate that a variety of anti-cancer agents are more effective in RhoJ-knockout mice, suggesting that inhibition of RhoJ signalling might have therapeutic benefit.

Recently, roles beyond angiogenesis have been identified for RhoJ. In melanoma cell lines, it was discovered that not only did RhoJ affect their motility and invasion (Ho et al., 2013), but that it also modulated chemoresistance by affecting DNA damage sensing (Ho et al., 2012). The latter required the activation of PAK1, and it will be of interest to discover whether the GIT–PIX complex is also involved. Understanding the biology of RhoJ might provide therapeutic opportunities in targeting both tumour cells and the tumour vasculature.

## MATERIALS AND METHODS

### Reagents

All chemicals were obtained from Sigma-Aldrich (Gillingham, UK) unless otherwise stated. For western blotting, mouse monoclonal anti-human RhoJ (Abcam, Cambridge, UK), mouse monoclonal anti-chicken tubulin (Sigma-Aldrich, Gillingham, UK); rabbit polyclonal anti-human GIT1 (Cell Signaling Technology, Hitchin, UK); mouse monoclonal anti-GFP (Clone 3E1, Cancer Research UK, London, UK); and rabbit monoclonal anti-phospho-GIT2 (Tyr392) (Cell Signaling Technology, Hitchin, UK) antibodies were used. For western blotting and immunofluorescence, rabbit polyclonal anti-human β-PIX (Millipore, Livingstone, UK); rabbit monoclonal anti-human GIT2 (Cell Signaling Technology, Hitchin, UK); purified anti-rabbit RhoJ polyclonal [as previously described (Kaur et al., 2011)] antibodies were used. For immunofluorescence mouse monoclonal anti-human vinculin (hVIN-1, Sigma-Aldrich, Gillingham, UK); rabbit polyclonal anti-human GIT1 (Santa Cruz Biotechnology, Santa Cruz, USA) antibodies were used. For immunofluorescence staining of tumour vessels anti-mouse CD31 (MEC13.3; BD Biosciences, Oxford, UK) antibody was used. Secondary antibodies were as follows: goat polyclonal anti-mouse immunoglobulin conjugated to horseradish peroxidase (HRP; Dako Cytomation, Ely, UK), donkey polyclonal anti-rabbit IgG conjugated to HRP (GE Healthcare), goat polyclonal anti-mouse IgG conjugated to Alexa Fluor 488 (Life Technologies, Paisley, UK), donkey polyclonal anti-rabbit IgG conjugated to Alexa Fluor 488 (Life Technologies, Paisley, UK), goat polyclonal anti-mouse IgG conjugated to Alexa Fluor 546 (Life Technologies, Paisley, UK), goat polyclonal anti-mouse IgG conjugated to Alexa Fluor 647 (Life Technologies, Paisley, UK) goat polyclonal anti-rat IgG conjugated to Alexa Fluor 546 (Life Technologies, Paisley, UK). Inhibitors used were FAK inhibitor PF 573228 (1 µM, SelleckChem, Munich, Germany); and Dasatinib (50 nM, SelleckChem, Munich, Germany).

### Plasmids and siRNA duplexes

The plasmids used for lentivirus production were psPAX2 (Lentiviral packaging for mammalian expression; Addgene, Cambridge, USA) and pMD2G (Envelope plasmid; Addgene, Cambridge, USA) in combination with pWPI (a lentiviral mammalian expression vector with an EMCV IRES-EGFP cassette; Addgene, Cambridge, USA) or pWPXL (Addgene, Cambridge, USA) for GFP expression or pWPXL-GFP-daRhoJ, constructed as previously described (Kaur et al., 2011). pWPXL-paxillin-RFP was constructed by subcloning the paxillin–RFP from pcDNA3.1-mRFP-N-paxillin (Parsons et al., 2008) into pWPXL and in the process removing the GFP cassette. For yeast two-hybrid experiments, daRhoJ (Q79L) or dnRhoJ (T35N) each lacking the C-terminal CAAX box were cloned as *Bam*HI-*Eco*RI fragments into the plasmid pGBT9 and expressed as fusion proteins with the Gal4p DNA-binding domain. GIT1, β-PIX, GIT1 amino acids 1–378 and GIT1 amino acids 1–258 where each cloned as *Eco*RI fragments into pACT2 to create Gal4p activation domain fusions. The yeast strain PJ69-4A, which comprises a GAL1::HIS3 promoter, was used as the host strain for the assays (Heath et al., 2004). The following siRNA duplexes were used: RhoJ siRNA, 5′-AGAAACCUCUCACUUACGAG-3′ (Eurogentec, Southampton, UK); GIT2 siRNA, 5′-CGAUGAAGUUGACAGGCGATT-3′; GIT1 siRNA, 5′-GGCAUUACAUCAUCCCACATT-3′; and β-PIX siRNA, 5′-CAGATAGACAAGATATTCATT-3′ (Life Technologies, Paisley, UK). Control siRNAs were from both Eurogentec (Southampton, UK) and Life Technologies (Paisley, UK). Alternative duplexes used for experiments in the supplementary data were as follows: β-PIX, 5′-CAACGACAGGAATGACAATTT-3′, GIT1, 5′-ACAUCUCCAUUGUCAAGCATT-3′; GIT2, 5′-CGUUGAUUAUGCAAGGCAATT-3′, and RhoJ, 5′-CCACUGUGUUUGACCACUAU-3′.

### Cell culture

Human umbilical vein endothelial cells (HUVECs) were used between passage 1 and 6 for all experiments. Umbilical cords were obtained from Birmingham Women's Health Care NHS Trust after delivery; mothers had given informed consent. HUVECs were cultured to confluence in Media 199 containing 4 mM L-glutamine, 90 µg/ml heparin, 10% (v/v) fetal bovine serum (PAA, The Cell Culture Co, Yeovil, UK) and purified bovine brain extract (Maciag et al., 1979). HEK293T cells and human dermal fibroblasts (Promocell, Heidelberg, Germany) were cultured in Dulbecco's modified Eagle's medium (DMEM) supplemented with 4 mM L-glutamine, penicillin-streptomycin solution and 10% (v/v) fetal bovine serum. Methods for siRNA transfection and lentiviral transduction are as described previously (Kaur et al., 2011). Transfections of HUVECs with siRNA duplexes were performed using RNAiMAX lipofectamine (Life Technologies, Paisley, UK) at a final concentration of 0.3% (v/v) with duplexes at 10 nM in OptiMEM. Lentivirus was generated in HEK293T through transient transfection with a combination of the packaging, envelope and expression plasmids listed above. The mixture of plasmids was incubated in OptiMEM (Life Technologies, Paisley, UK) with polyethylenimine (Sigma-Aldrich, Gillingham, UK) at 36 µg/ml for 10 minutes at room temperature prior to adding to HEK293T cells in their normal growth medium. Medium containing virus was harvested 48 hours after transfection, passed through a 0.45 µm^2^ pore syringe filter (Corning, Amsterdam, The Netherlands), supplemented with 8 µg/ml polybrene (Sigma-Aldrich, Gillingham, UK) and endothelial growth supplements: bovine brain extract and heparin as described above, and incubated with the HUVECs to be transduced.

### Generation of the RhoJ-knockout mouse and tumour implantation assays

Mice were housed at the Birmingham Biomedical Services Unit (Birmingham, UK), animal maintenance and experimentation had appropriate Home Office approval and licensing. C57BL/6N JM8.N4 feeder-independent embryonic stem cells containing the RhoJ-knockout first promoter driven cassette [RhoJtm1a(KOMP)Wtsi project ID CSD25401] were procured from the Knockout Mouse Project (University of California, Davis, USA) and knockout mice were generated by the Transgenic Mouse Facility at the University of Birmingham. Chimeric mice were generated by injection of embryonic stem cells into albino C57BL/6 mice and were bred to C57BL/6 females to generate mice heterozygous for the cassette. These mice were crossed with PGK-Cre mice, which constitutively express Cre recombinase (Lallemand et al., 1998) resulting in the removal of the LoxP flanked exon 2 of RhoJ. To induce tumour growth, 10^6^ Lewis lung carcinoma cells were injected subcutaneously into the flank of male mice at 8–9 weeks of age. After 2 weeks, tumour mass was determined and then the tumours were frozen in OCT compound and serial sections cut at 6 µm.

### Preparation of lysates, GFP-trap experiments and western blotting

All cell lysates were prepared using Rho-assay lysis buffer (1% (v/v) NP40, 1% (w/v) N-octyl-β-D-glucopyranoside, 25 mM HEPES pH 7.5, 30 mM MgCl_2_, 150 mM NaCl, mammalian protease inhibitor cocktail, 10 mM NaF, 2 mM NaVO_3_). Cells were resuspended in lysis buffer on ice for 15 minutes prior to spinning at 21,910 ***g*** for 10 minutes at 4°C. For western blotting of cell lysates, lysates were mixed with sample buffer and subjected to SDS-PAGE. For GFP-trap experiments 2×10^7^ HUVECs were plated the day prior to the experiment, they were lysed with 1 ml Rho-assay lysis buffer, 25 µl of the lysate was mixed with an equal volume of 2× SDS-PAGE sample buffer, and the remainder was incubated with 20 µl washed GFP-trap A beads (Chromotek, Planegg-Martinsried, Germany) for 1 hour at 4°C with rotation. Subsequently, beads were washed three times with Rho-assay lysis buffer and bound proteins eluted with 50 µl 2× SDS-PAGE sample buffer. Samples were then subjected to SDS-PAGE electrophoresis and western blotting. SDS-PAGE and western blotting were performed using standard techniques. Primary antibodies used were as indicated with HRP-conjugated secondary antibodies, they were developed with ECL western blotting detection reagents (GE Healthcare).

### Yeast two-hybrid

Yeast media and culture conditions were as previously described (Sherman, 1991). The strain PJ69-4A (MATa trp1-901, leu2-3,112, ura3-52, his3-200, gal4Δ, gal80Δ, GAL2-ADE2, LYS2::GAL1-HIS3, met2::GAL7-lacZ) (James et al., 1996) was transformed with combinations of GBT9 and pACT2 plasmids (described above) using the lithium acetate method (Gietz and Woods, 2002). Yeast two-hybrid assays were performed as previously described (Heath et al., 2004). Briefly, cultures were grown in selective medium to stationary phase, diluted to a OD600 of 0.5 and spotted on to synthetic medium either containing or lacking histidine, plates lacking histidine also contained amino-1,2,4-triazole (3-AT) at 3 mM. Plates were incubated for 3 to 5 days at 30°C.

### Immunofluorescence staining

The protocol for immunofluorescence staining is as described previously (Kaur et al., 2011). For all staining other than with anti-RhoJ antisera, cells were fixed with 4% (w/v) paraformaldehyde in PBS for 15 minutes, neutralised with 50 mM NH_4_Cl in PBS for 10 minutes and then permeabilised with 0.1% (v/v) Triton X-100 in PBS for 4 minutes, with washing in PBS between each step. Blocking was performed using 3% (w/v) BSA, 10% (v/v) FCS, 0.1% (v/v) Tween-20, 0.01% (w/v) sodium azide in PBS for 1 hour at room temperature. For RhoJ staining, cells were fixed and permeabilized with ice-cold methanol for 5 minutes prior to blocking for 1 hour at room temperature in 4% (w/v) BSA in PBS. Primary and secondary antibodies were diluted in the blocking buffer and cells were mounted onto slides using ProLong Gold Antifade reagent with DAPI (Life Technologies, Paisley, UK) and left in the dark overnight. Staining was analysed using the Zeiss LSM 510-UV confocal microscope, and imported using the LSM Image Brower (Zeiss, Cambridge, UK). In order to visualise tumour vessels, tissue was fixed with 4% paraformaldehyde and blocked with 2.5% normal horse serum (Vector Laboratories, Peterborough, UK). Immunostaining was performed using 1.5 µg/ml anti-mCD31 (MEC13.3) (BD Biosciences, Oxford, UK) and 5 µg/ml anti-rat IgG conjugated to Alexa Fluor 546 (Life Technologies Paisley, UK). Images were taken using an Axioskop2 microscope and AxioVision SE64 Rel4.8 software (Zeiss, Cambridge, UK).

### TIRF microscopy

TIRF microscopy was used to monitor focal adhesion turnover. HUVECs were transduced to express paxillin–RFP, and manipulated for their expression of RhoJ as indicated. A day before imaging, cells were replated onto 35-mm diameter, 20 mm microwell MatTek plates (No. 1.5 uncoated coverslip, MatTek Corporation, Ashland, USA). The next day and 2–4 hours before imaging, a scratch wound was made in the monolayer with a sterile 20 µl pipette tip and migrating cells at the scratch edge were monitored by timelapse TIRF microscopy. Prior to imaging, media was replaced with DMEM lacking phenol red (Sigma-Aldrich, Gillingham, UK), supplemented as described above for HUVEC media. Cells at the edge were selected and monitored for 1.5 h using a Nikon TIRF system on a Nikon Eclipse Ti inverted microscope (Nikon, Surrey, UK) at 37°C with CO_2_ buffering. Cells were imaged every 2 minutes using a Green Diode 561 nm laser and a CFL Plan Apo 60× NA 1.49 objective. Images were captured on a 12-bit Ixon 1M EMCCD camera controlled by Nikon NIS Elements software.

### Image analyses

In order to assess focal adhesion turnover, turnover times were measured from 10–15 adhesions from each of 11–12 cells (three or four cells from three independent experiments, a total of 155 or 165 adhesions per condition). Focal adhesions were manually tracked using the Cell Counter plug-in for ImageJ. There were no statistically significant differences using the Kruskal–Wallis test between data from each cell within experimental groups and so data for each focal adhesion within an experimental group were pooled. Turnover durations were recorded from the point at which the adhesion was first visible, to the point where they could no longer be seen. To assess assembly and disassembly, these same adhesions were manually outlined in ImageJ and mean grey values measured for each frame they were visible. The duration of assembly was the point from the first appearance of the adhesion to the time when the highest mean grey value was recorded. Disassembly was from this brightest point, to when the adhesion was no longer visible. To determine levels of recruitment of GIT1, GIT2, RhoJ or β-PIX, vinculin-stained focal adhesions distributed around the perimeter of the cell were selected. These were manually outlined on ImageJ. Mean grey values were measured and recorded for staining against GIT1, GIT2, RhoJ or β-PIX. The mean grey value for the protein of interest was only measured after selection and drawing round the vinculin-positive adhesions to eliminate any bias. For each experimental replicate, all data points were scaled to the mean of the mean grey value for the control siRNA for that experiment, which was set to 100. This enabled the data from each experimental replicate to be combined. To assess focal adhesion size, cells were stained with anti-vinculin antibodies and the focal adhesion size was measured as the area of fluorescence. For GFP- or GFP–daRhoJ-expressing cells, 15 focal adhesions from three cells per condition from three independent experiments were manually outlined on ImageJ, and areas measured. For cells transfected with control siRNA or RhoJ siRNA, at least 20 adhesions were measured per cell. In order to analyse vessel density, images from the red channel were inverted using ImageJ and vessel numbers from each field were determined using AngioSys software (Cellworks, Buckingham, UK). For each tumour the mean vessel density was derived from three or four fields of view.

### Tube-forming assays

Natural Matrigel (VWR, East Grinstead, UK) was thawed overnight on ice at 4°C. The wells of a 12 well plate were wetted with PBS prior to adding 70 µl of matrigel. The basement membrane extract was allowed to solidify at 37°C for 30 minutes. Cells were harvested and seeded at a density of 1.4×10^5^/well in HUVEC media. Cells were then incubated at 37°C with 5% CO_2_ for a further 24 hours. Tube formation was observed by taking pictures using Leica DM IL microscope and USB 2.0 2M Xli camera (Leica Microsystems, Houston, USA) and five or six images were captured per condition. These were analysed using the angiogenesis analyser plugin for ImageJ [Carpentier G., Angiogenesis Analyzer for ImageJ (2012) available online: http://imagej.nih.gov/ij/macros/toolsets/Angiogenesis%20Analyzer.txt, accessed February 2014], the loop number is given by the mesh parameter. For each experiment the mean loop number per condition at 12 and 24 hours was calculated from five or six images, and the mean used for each experimental replicate.

### Statistical analyses

All experiments were performed at least three times with similar results. To compare datasets for focal adhesion assembly and disassembly, RhoJ, GIT1, GIT2 and β-PIX recruitment and tumour size and vascular density the Mann–Whitney test was performed. To analyse focal adhesion area and matrigel loop number data sets, the Student's *t*-test was used. All calculations were performed using the Prism software (GraphPad, La Jolla, USA).

## Supplementary Material

Supplementary Material
