# Supplementary material for: RhoJ interacts with the GIT–PIX complex and regulates focal adhesion disassembly
Source: J Cell Sci. 2014 Jul 15;127(14):3039–51. doi: 10.1242/jcs.140434 (PMC4106786; doi:10.1242/jcs.140434)
Supplement: Supplementary Material [file supp_127_14_3039__index.html]

RhoJ interacts with the GIT–PIX complex and regulates focal adhesion disassembly — Supplementary Material 

# RhoJ interacts with the GIT–PIX complex and regulates focal adhesion disassembly

## JCS140434 Supplementary Material

**Files in this Data Supplement:**

- **Supplementary Material**
