# Supplementary material for: RhoJ interacts with the GIT–PIX complex and regulates focal adhesion disassembly
Source: J Cell Sci. 2014 Jul 15;127(14):3039–51. doi: 10.1242/jcs.140434 (PMC4106786; doi:10.1242/jcs.140434)
Supplement: Supplementary Material [file supp_127.14.3039_JCS140434.pdf]

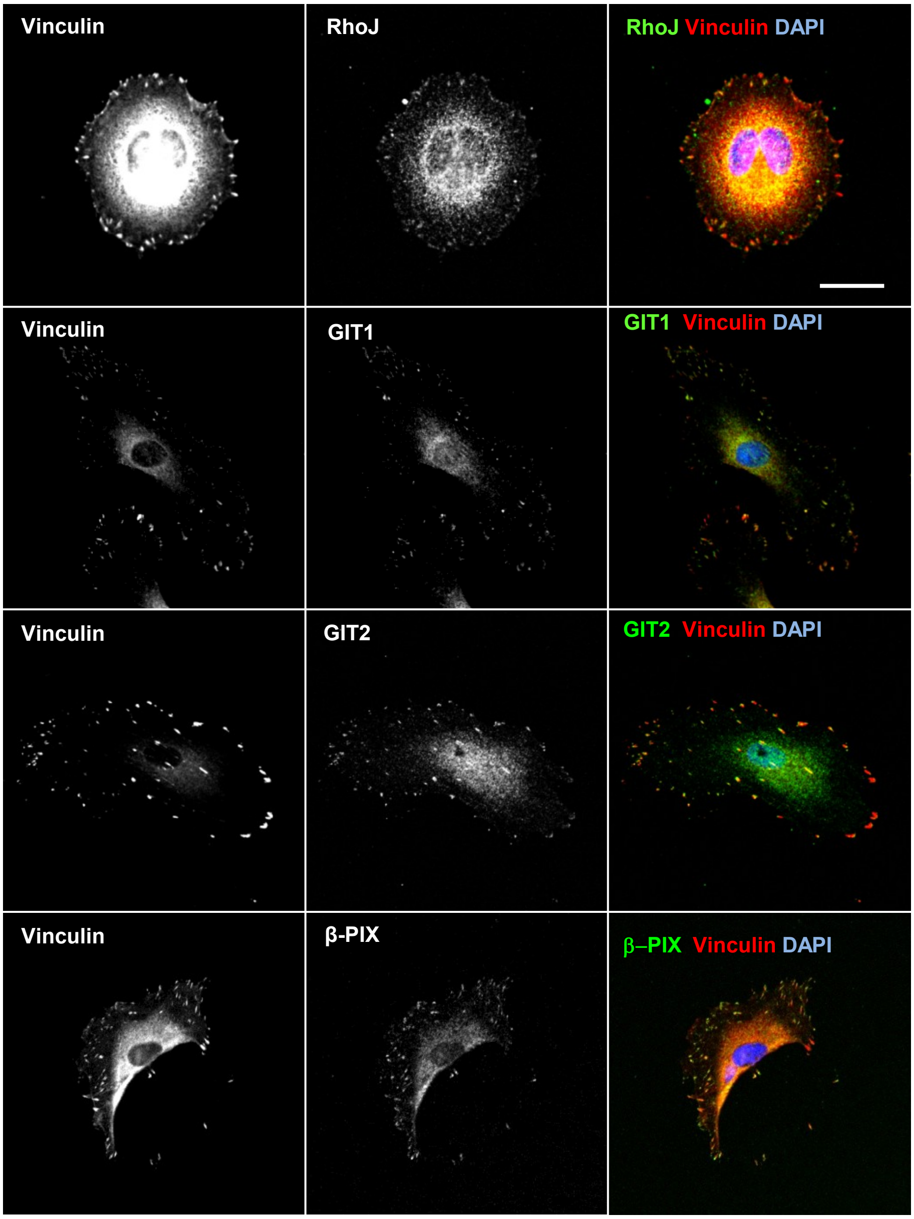

Figure S1. RhoJ, GIT1, GIT2 and β-PIX localize to focal adhesions and reducing RhoJ expression reduces RhoJ size
(A) HUVECs were fixed and stained for vinculin and RhoJ, GIT1, GIT2 or β-PIX. Scale bar: 20 μm

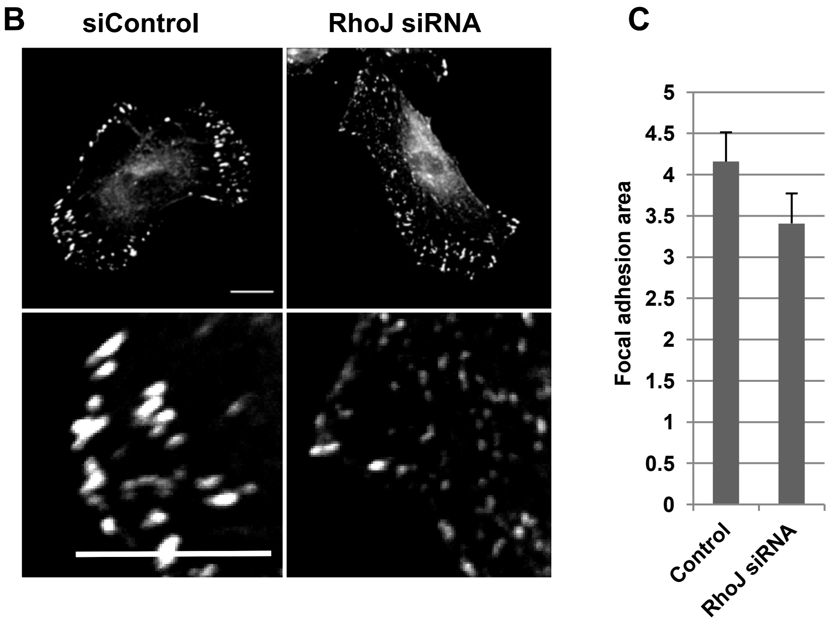

Figure S1. RhoJ, GIT1, GIT2 and β-PIX localize to focal adhesions and reducing RhoJ expression reduces RhoJ size

(B) HUVECs were transfected with siControl or an alternative RhoJ siRNA duplex and after 48 hours were stained with vinculin specific antibodies. The box indicates the enlarged area. Scale bar: 20 µm. **(C)** Focal adhesion areas were measured using ImageJ of 100 - 120 focal adhesions from a total of 5 or 6 cells and mean focal adhesion area calculated per condition. This was performed three times and plotted are the mean focal adhesion areas from each experiment, error bar represent SEM. A reduction in focal adhesions area was observed in each of the three experiments.

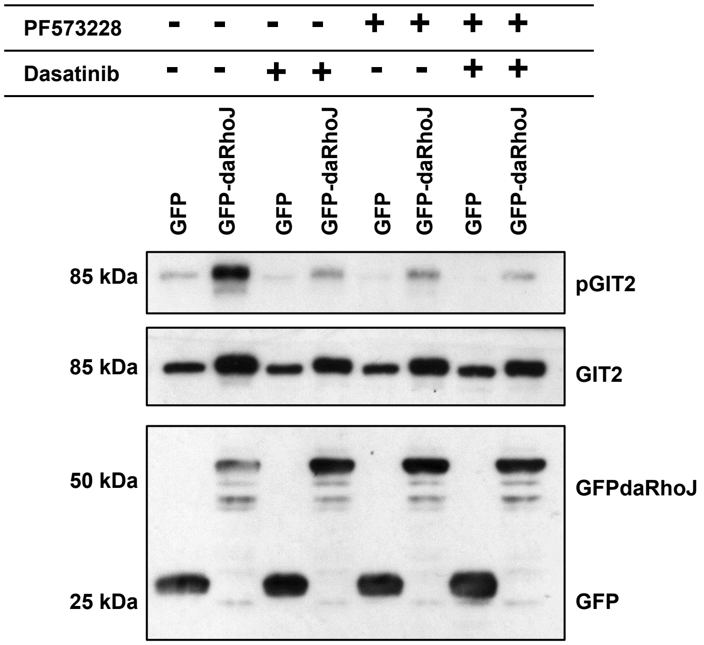

Figure S2. Inhibition of Src and FAK reduce levels of phospho-GIT2 (Y392)
HUVECs were transduced to express either GFP or GFP-daRhoJ. Prior to lysis they were incubated with 50 nM Dasatinib (Src inhibitor), 1 μM PF573228 (FAK inhibitor) or both. Lysates were subjected to SDS-PAGE and western blotting for phosph-GIT2 (Y392), GIT2 or GFP. This is representative of 3 experiments.

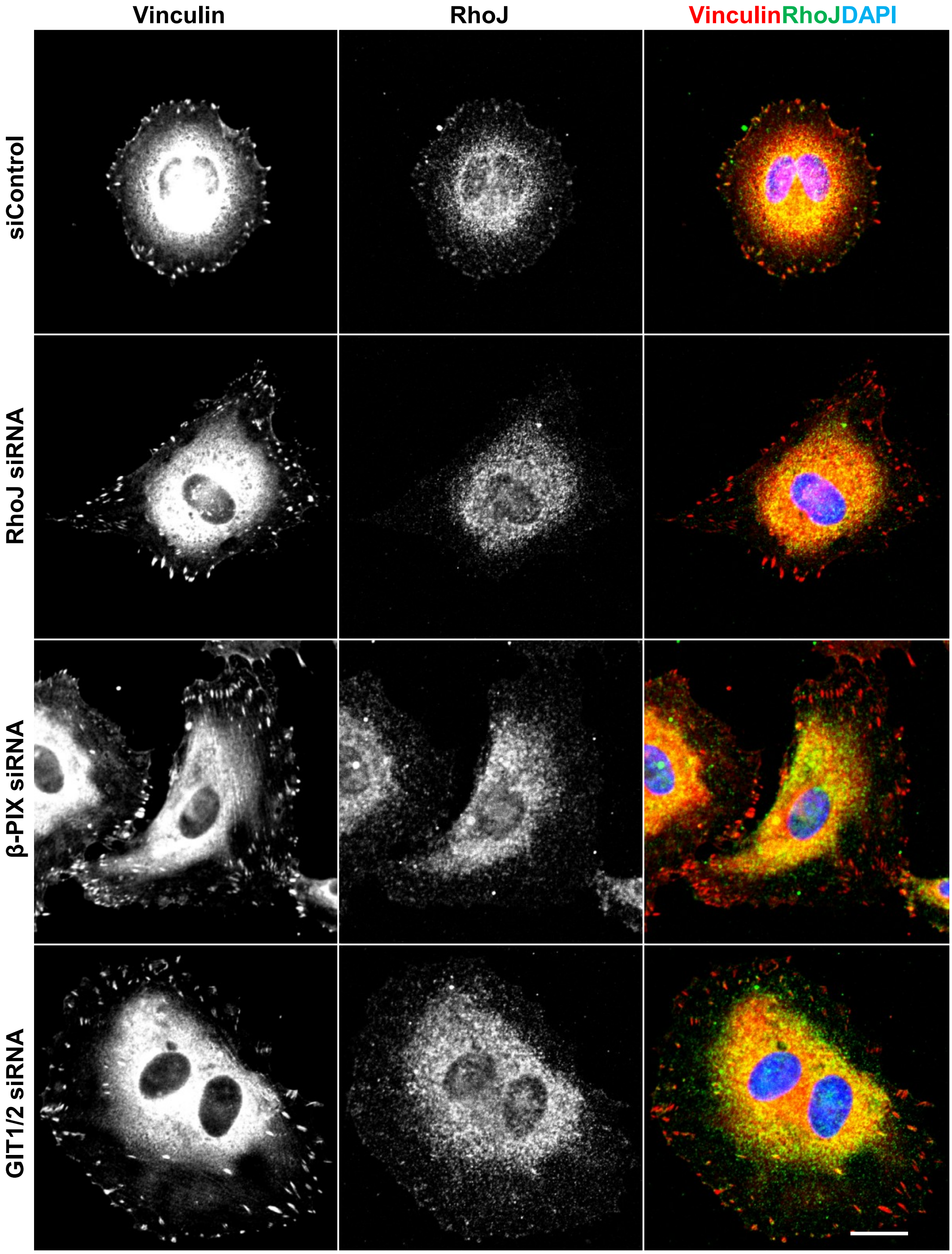

Figure S3(A) GIT1/2 and β-PIX knockdown reduce RhoJ recruitment to focal adhesions. HUVECs were transfected with siControl, RhoJ siRNA, β-PIX siRNA or both GIT1 and GIT2 siRNA duplexes. After 48 hours HUVECs were fixed and stained for vinculin and RhoJ. Scale bar: 20 μm

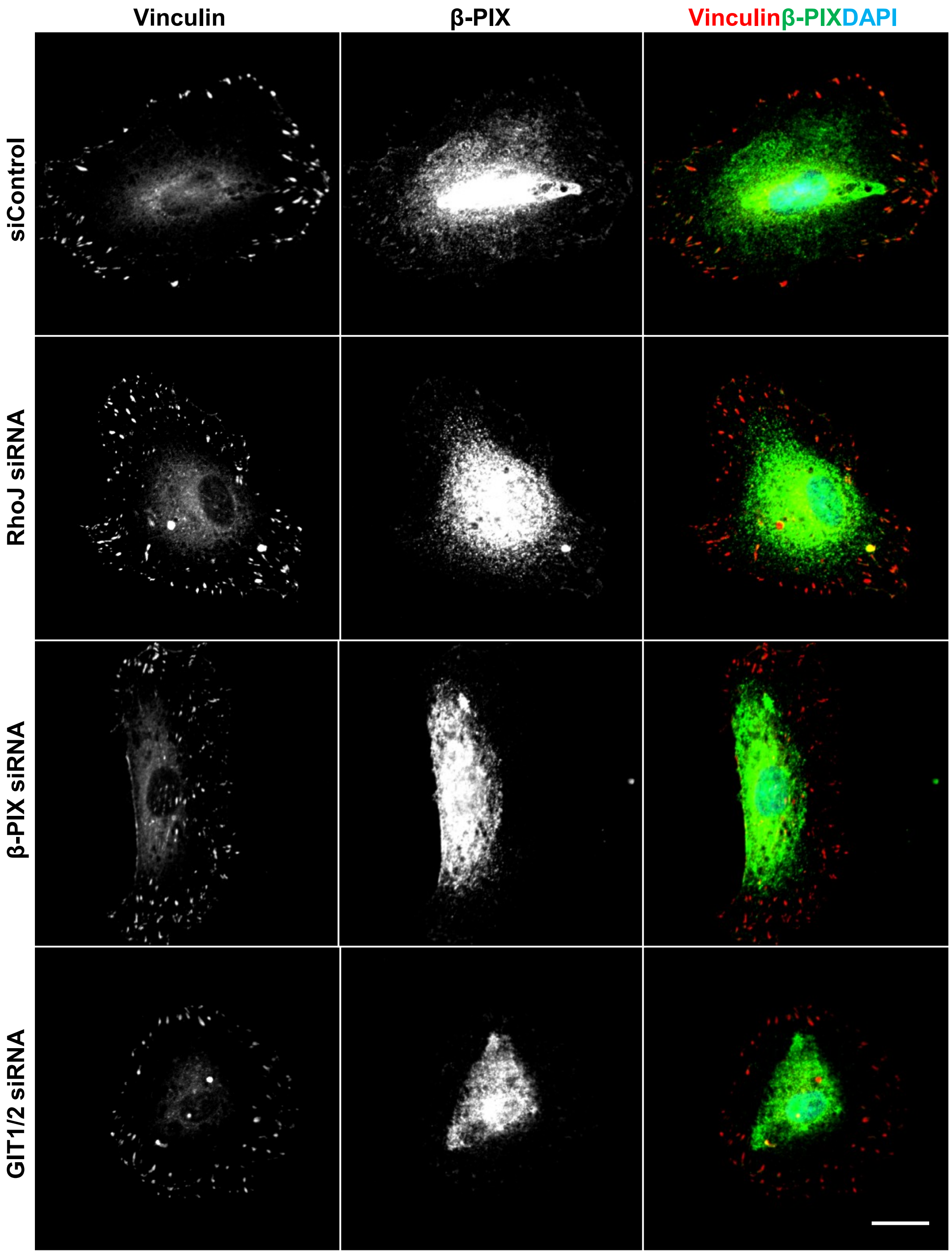

Figure S3(B) GIT1/2 and RhoJ knockdown reduce β-PIX recruitment to focal adhesions. HUVECs were transfected with siControl, RhoJ siRNA, β-PIX siRNA or both GIT1 and GIT2 siRNA duplexes. After 48 hours HUVECs were fixed and stained for vinculin and β-PIX. Scale bar: 20 µm

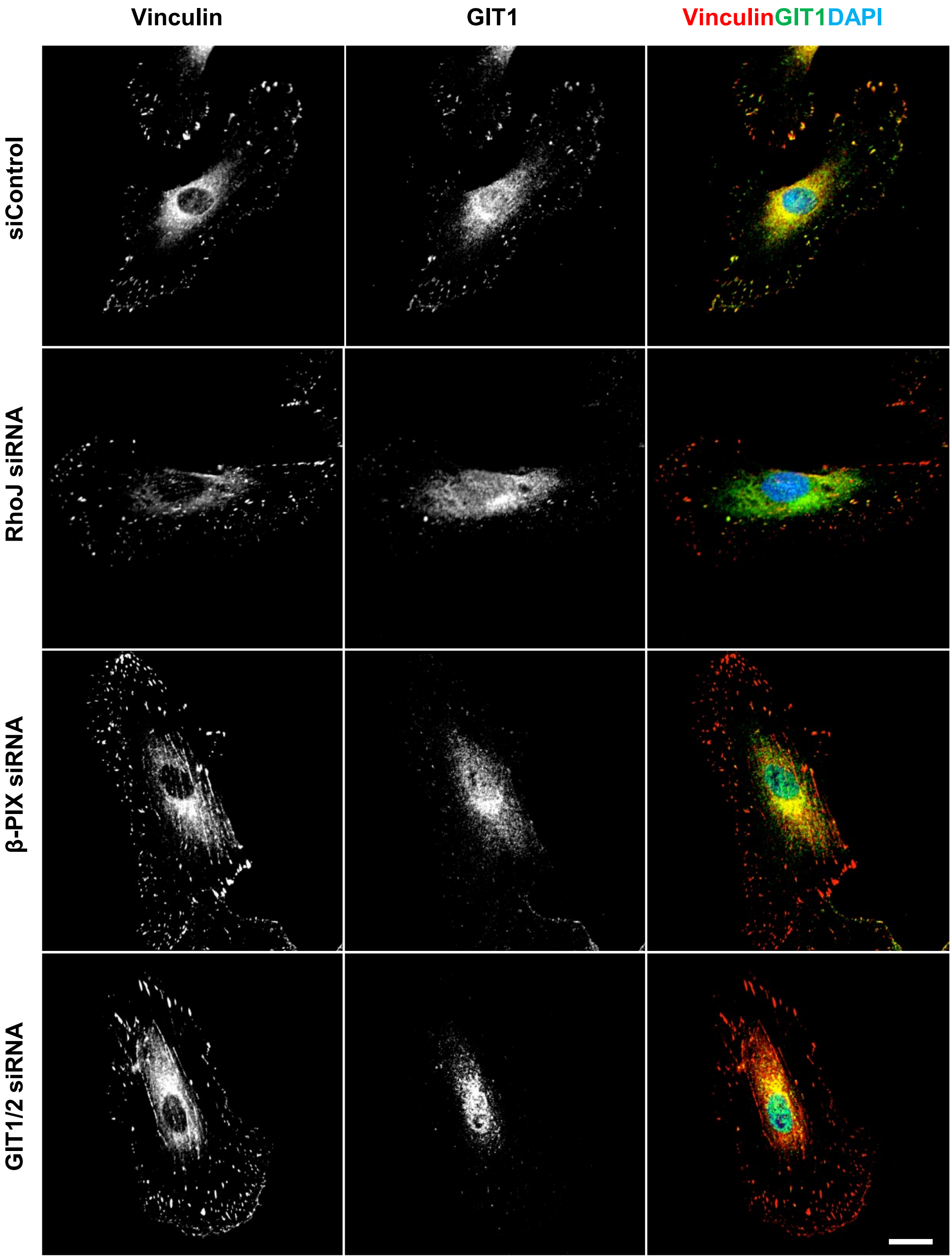

Figure S3(C) β-PIX and RhoJ knockdown reduce GIT1 recruitment to focal adhesions. HUVECs were transfected with 10 nMsiControl, RhoJ siRNA, β-PIX siRNA or both GIT1 and GIT2 siRNA duplexes. After 48 hours HUVECs were fixed and stained for vinculin and GIT1. Scale bar: 20 μm

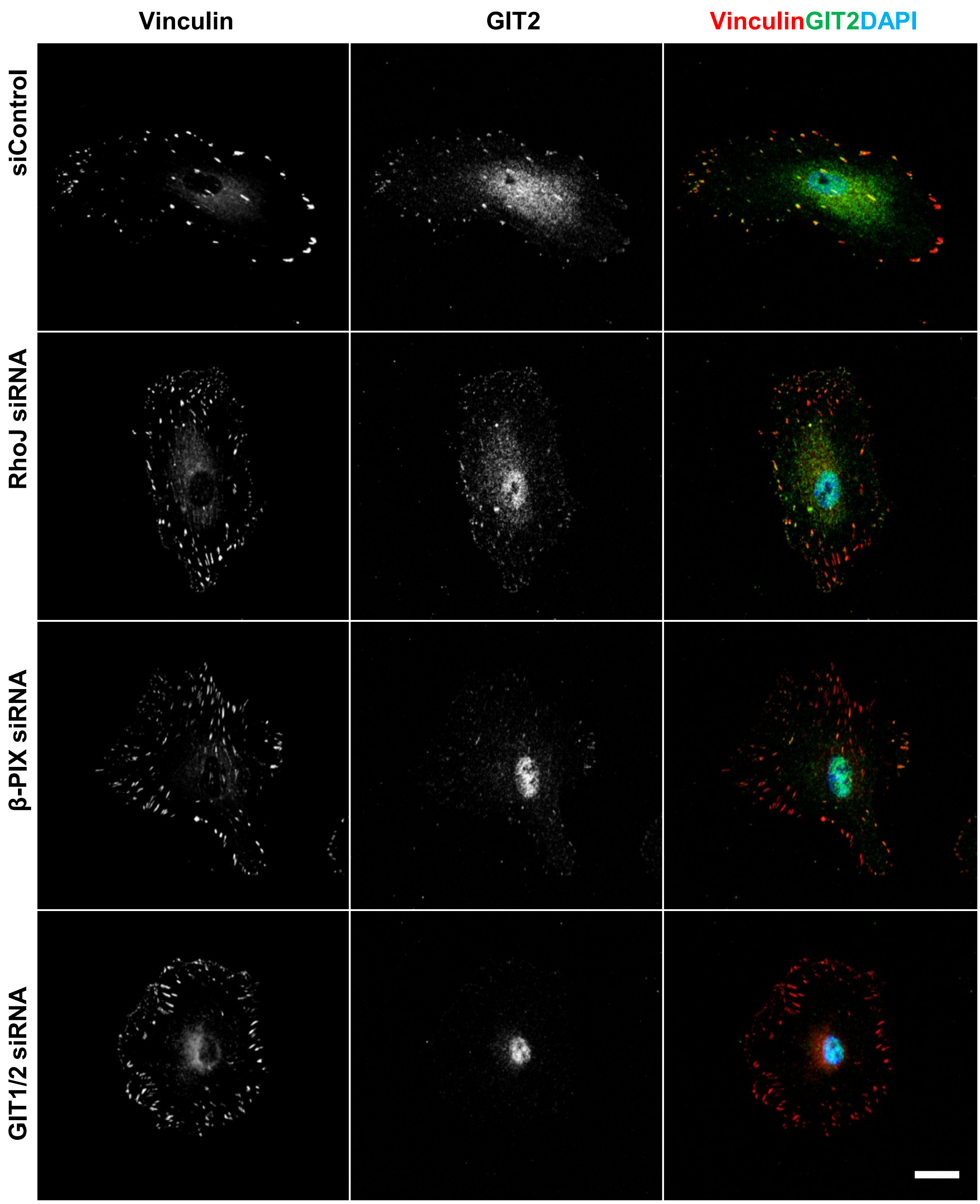

Figure S3(D) β-PIX and RhoJ knockdown reduce GIT2 recruitment to focal adhesions. HUVECs were transfected with siControl, RhoJ siRNA, β-PIX siRNA or both GIT1 and GIT2 siRNA duplexes. After 48 hours HUVECs were fixed and stained for vinculin and GIT2. Scale bar: 20 μm

E

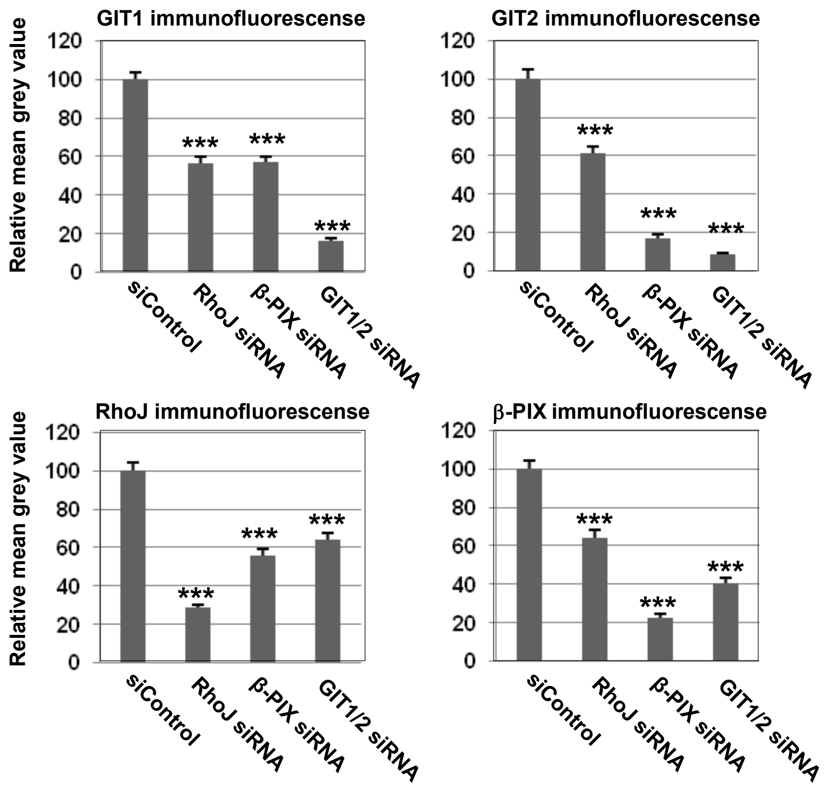

Figure S3(E). Reciprocal regulation of the recruitment of RhoJ, GIT1/2 and β-PIX to focal adhesions.
HUVECs were transfected with siControl, and alternative duplexes specific for RhoJ, β-PIX and both GIT1 and GIT2. After 48 hours HUVECs were fixed and stained for vinculin and either RhoJ, β-PIX, GIT1 or GIT2. For each experiment, the mean grey value of either GIT1, GIT2, RhoJ or β-PIX staining (as indicated) was calculated for 20 adhesions per cell from 3 cells using ImageJ according the materials and methods. For each replicate experiment all data points were scaled to the mean of the siControl which was set at 100. Plotted is the mean of the all scaled data points from each condition from each of the experimental replicates, error bar represent SEM, *** indicates $p<0.001$ by a Mann Whitney test comparing each of the data points to the siControl.

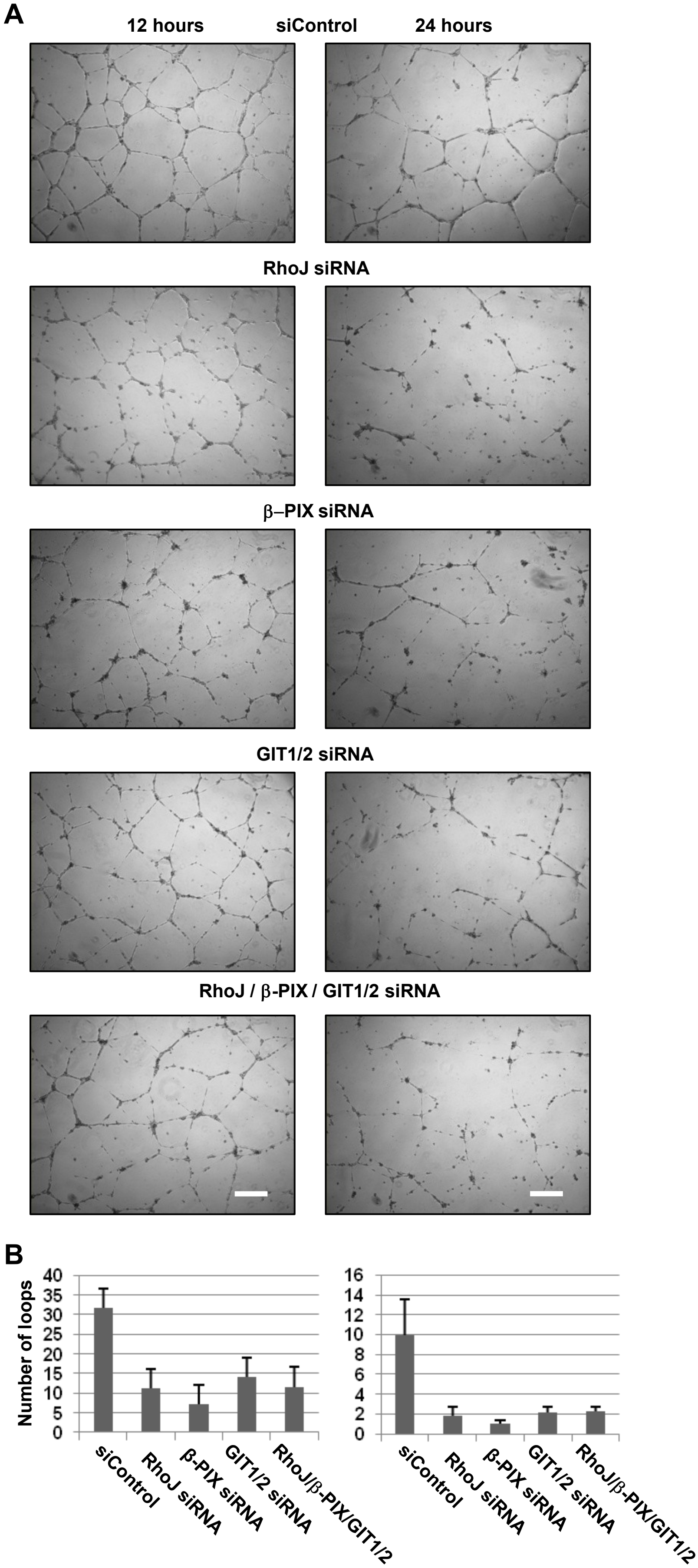

Figure S4. Knockdown of RhoJ, β-PIX, GIT1/2 or a combination of all four similarly impairs tube formation. **(A)** HUVECs were transfected with siControl, and alternative RhoJ siRNA, β-PIX siRNA or GIT1 and GIT2 siRNA or a combination of the alternative RhoJ, β-PIX, GIT1 and GIT2 duplexes. 48 hours after transfection, the cells were replated on matrigel and imaged after 12 and 24 hours. Scale bar: 200 µm **(B)** Analysis of the tubule formation using the angiogenesis analyser ImageJ plugin to show the number of loops formed by the tubules. For each experiment the mean loop number was calculated from five to six fields of view. The mean of these values from three replicates is plotted.
